# Instant disembodiment of virtual body parts

**DOI:** 10.3758/s13414-022-02544-w

**Published:** 2022-08-31

**Authors:** Julia Eck, David Dignath, Andreas Kalckert, Roland Pfister

**Affiliations:** 1 Department of Psychology III, Julius-Maximilians-Universität Würzburg, Röntgenring 11, 97070 Würzburg, Germany; 2 Department of Psychology, Eberhard Karls University of Tübingen, Tübingen, Germany; 3 Cognitive Neuroscience and Philosophy, University of Skövde, Skövde, Sweden

**Keywords:** Body representation, Embodiment, Disembodiment, Moving rubber hand illusion, Virtual hand illusion

## Abstract

Evidence from multisensory body illusions suggests that body representations may be malleable, for instance, by embodying external objects. However, adjusting body representations to current task demands also implies that external objects become disembodied from the body representation if they are no longer required. In the current web-based study, we induced the embodiment of a two-dimensional (2D) virtual hand that could be controlled by active movements of a computer mouse or on a touchpad. Following initial embodiment, we probed for disembodiment by comparing two conditions: Participants either continued moving the virtual hand or they stopped moving and kept the hand still. Based on theoretical accounts that conceptualize body representations as a set of multisensory bindings, we expected gradual disembodiment of the virtual hand if the body representations are no longer updated through correlated visuomotor signals. In contrast to our prediction, the virtual hand was instantly disembodied as soon as participants stopped moving it. This result was replicated in two follow-up experiments. The observed instantaneous disembodiment might suggest that humans are sensitive to the rapid changes that characterize action and body in virtual environments, and hence adjust corresponding body representations particularly swiftly.

## Introduction

The experience of having a “self” is intrinsically linked to the experience of having a body. Our bodies enable us to interact with our environment and to perceive ourselves as individual agents (Gallagher, 2000). A physical body, however, is limited to interactions within the physical world, whereas modern technology also enables interactions in virtual environments.

How does the “self” adapt to such virtual interactions? Evidence from experiments using multisensory body illusions suggests that body representations are not set in stone but can be flexibly adjusted based on recent multisensory experiences (e.g., Botvinick & Cohen, 1998; Ehrsson et al., 2004; Makin et al., 2008; Tsakiris & Haggard, 2005).

In a seminal study, participants were seated with both hands resting on a table in front of them. One hand was hidden behind a screen, while a rubber hand was placed in an anatomically plausible position in front of the participant (Botvinick & Cohen, 1998). The real occluded hand and the rubber hand were stroked with a paintbrush either simultaneously (synchronous condition) or by introducing a short time delay (asynchronous condition). After participants experienced several minutes of synchronous visuotactile stimulation, they reported feeling as if the rubber hand was their own hand. This feeling was absent in the asynchronous condition. The authors suggested that ownership of the rubber hand occurred in the synchronous but not in the asynchronous condition because visual, tactile, and proprioceptive signals were correlated only in the former but not in the latter. The demonstration of the rubber hand illusion shows that the formation of body representations is based on integrating correlated multisensory signals (e.g., Ehrsson et al., 2004; Makin et al., 2008; Tsakiris & Haggard, 2005).

Feelings of embodiment of an artificial hand – i.e., the illusory experience that the rubber hand is a part of the person’s own body – have been replicated for different combinations of correlated multisensory signals (e.g., Dummer et al., 2009; Ehrsson et al., 2005; Kalckert & Ehrsson, 2012, 2014) and for virtual instead of artificial physical limbs (e.g., Kokkinara & Slater, 2014; Perez-Marcos et al., 2009; Sanchez-Vives et al., 2010). Furthermore, the use of augmented and virtual reality techniques also enabled full-body illusions, which aim at experimentally manipulating the representation of the whole body (e.g., Lenggenhager et al., 2007; Maselli & Slater, 2013; Petkova & Ehrsson, 2008). Based on results from partial and full body illusions, three main components of embodiment have been suggested: the experience of the body and its parts as belonging to oneself (self-identification or body ownership), the experience of the self and the body as being at the same location (self-location), and the experience that the world is perceived from the viewpoint of the owned body (first-person perspective; Blanke, 2012; Blanke & Metzinger, 2009; Maselli & Slater, 2013).

In partial body illusions, congruent multisensory stimulation seems to be necessary for the embodiment of the artificial body part (Botvinick & Cohen, 1998; Kalckert & Ehrsson, 2014; Tsakiris & Haggard, 2005). However, the embodiment of a whole virtual body can occur only by looking at a plausible virtual body even without the experience of correlated tactile or motor signals (Maselli & Slater, 2013).

Thus, if embodiment of external objects is regarded as a continuum from low anatomical plausibility, as in the case of body-external tools, to high anatomical plausibility, as in the case of embodiment for realistic virtual bodies, then the need for congruent multisensory stimulation to induce the illusion should increase as the anatomical plausibility of the external object decreases (Maselli & Slater, 2013; Samad et al., 2015). This assumption of an interaction between top-down and bottom-up processing is supported by two types of evidence. First, a humanoid shape and an anatomically plausible position are critical constraints for the embodiment of external physical or virtual objects (Petkova & Ehrsson, 2008; Tsakiris et al., 2010; Tsakiris & Haggard, 2005). Second, for tools and other non-corporeal objects, embodiment can occur despite low anatomical plausibility of these objects (Liesner et al., 2021; Maravita et al., 2003; Schettler et al., 2019). However, for embodiment to emerge for anatomically less plausible objects or non-corporeal objects, congruent multisensory stimulation must be supplemented by the experience of actively controlling the object (agency; e.g., Asai, 2016; Brugada-Ramentol et al., 2019; Cardinali et al., 2009; Kirsch et al., 2016; Liesner, Kirsch, Pfister, & Kunde, 2020b; Ma & Hommel, 2015a, 2015b; Short & Ward, 2009). Moreover, findings from several studies suggest that not only the current experience of controlling a non-corporeal object but also experience of agency with it in the past and/or prior knowledge about the object’s functional properties can enhance the strength of embodiment (Bassolino et al., 2010; Cardinali et al., 2021; Liepelt et al., 2017; Serino et al., 2007).

Whether agency influences the strength of embodiment in more realistic body illusions is still debated. In the moving rubber hand illusion, instead of passively receiving strokes on the real hand and the corresponding rubber hand, a mechanical connection between the real hand and the rubber hand enables participants to move either the whole rubber hand or only selected fingers (Dummer et al., 2009; Kalckert & Ehrsson, 2012, 2014, 2017). With this setup, direct comparisons between active and passive conditions of visuomotor stimulation (synchronous or asynchronous) are possible. While in the active condition participants perform movements by themselves, in the passive condition, movements are realized by the experimenter who moves the participants’ hand or finger. Although the sensory experience in the active and passive conditions is the same, the two conditions differ with respect to motor control processes or agency, which is present only in the active condition. Thus, comparing active and passive movements allows for inferences about whether agency contributes to the strength of embodiment. While some studies found evidence for a stronger illusion in the active synchronous as compared to the passive synchronous condition (Jenkinson & Preston, 2015; Kalckert & Ehrsson, 2017), others did not (Kalckert & Ehrsson, 2012, 2014; Tsakiris et al., 2006). The mixed evidence concerning the role of agency might suggest that for the embodiment of an object that resembles a real body part, such as the rubber hand, agency is less important than for the embodiment of non-corporeal objects. However, for the embodiment of non-corporeal objects, the past or present experience of agency with this object appears to be critical (e.g., Bassolino et al., 2010; Cardinali et al., 2009; Cardinali et al., 2021; Liepelt et al., 2017; Liesner et al., 2021; Maravita et al., 2003; Schettler et al., 2019).

Taken together, the available evidence suggests that humans embody external objects that mediate their actions in the physical or virtual world. Yet, understanding how body representations are expanded addresses only one side of the coin. Tuning body representations to current situational demands also requires that parts become detached – or disembodied – from the body representation if they are no longer warranted. Despite considerable research that contributed to a better understanding of the formation of body representations, little is known about disembodiment (De Vignemont, 2011). However, three lines of research on disembodiment have recently emerged.

### Studying disembodiment

One approach to study disembodiment under experimental conditions is to measure whether the real hand is disembodied during the rubber hand illusion. This method is based on the theoretical proposal that a “default” body model constrains what possibly can be experienced as belonging to the body and what cannot (Tsakiris, 2010; Tsakiris et al., 2010). According to this view, the real hand becomes disembodied during the rubber hand illusion because embodiment can be experienced for only two hands at a time (Longo et al., 2008; Moseley et al., 2008). Whether such constraints do indeed lead to disembodiment of the real hand has been debated (e.g., Folegatti et al., 2009). For instance, observations of supernumerary limb illusions (Ehrsson, 2009; Newport et al., 2010) suggest that more than two hands can be embodied at the same time without any accompanying experience of disembodiment (see also De Vignemont, 2011, for a discussion).

A second line of research is based on evidence from neurological patients. For example, in the case of asomatognosia, after brain lesions, patients typically suffer from feelings of disembodiment of their contralesional limb (mostly their left hand, following lesions in the right fronto-temporo-parietal cortex). It has been suggested that the default bodily self-perception is impaired in these patients because of deficits in multisensory integration (Vallar & Ronchi, 2009). This assumption is also supported by findings on experimentally induced disembodiment in healthy participants (Gentile et al., 2013; Lesur et al., 2020; Newport & Gilpin, 2011). Two of these studies used a mixed-reality setup. Through a head-mounted display, participants saw either their own arm, which was video recorded prior to the experiment (Gentile et al., 2013), or their whole body, which was recorded and transmitted during the experiment (Lesur et al., 2020). Moreover, in both studies, participants saw the presented body or body parts from a first-person perspective and at an anatomically plausible position. In a third study, participants placed their hands in a multisensory illusion box where the visibility and the location of the hand could be manipulated (Newport & Gilpin, 2011). Because these studies used projections of the participants’ real body or body parts, a high degree of self-identification with the virtual counterpart could be presupposed. This allowed controlled investigation of the factors underlying the disembodiment of the own body or body parts. By providing visual information about the body that was incongruent with the corresponding tactile or sensorimotor sensations, the experience of a multisensory mismatch was triggered. The induced multisensory conflict led to disembodiment of one’s own body or body part (Gentile et al., 2013; Lesur et al., 2020; Newport & Gilpin, 2011). These findings suggest that disembodiment of a body part or even the whole body is based on the disintegration of visual, proprioceptive, and tactile or sensorimotor signals.

The studies described so far have focused on disembodiment of one’s own physical body parts. In contrast, a third approach to studying disembodiment was used in a recently proposed paradigm that focused on disembodiment of a previously embodied rubber hand (Pfister et al., 2021; for a similar design, see Abdulkarim et al., 2021). This study aimed to distinguish between two theoretical models of disembodiment: a *persistence* model and an *updating* model. Based on findings such as feelings of phantom limbs after amputation (e.g., Ramachandran & Rogers-Ramachandran, 1996), the persistence model assumes that an embodied entity remains within the body representation unless new sensory information actively contradicts the current state of embodiment of the given body part. According to the updating model, embodiment emerges from multisensory bindings that dissolve over time if they are not renewed continuously (e.g., Blanke, 2012; Samad et al., 2015). The general logic of the experiment was to conduct a moving rubber hand illusion first, which then allowed for observing how embodiment of the artificial hand dissolves in different conditions. Each trial consisted of a 2-min embodiment phase and a 2-min disembodiment phase. Every 30 s, participants rated their sense of embodiment of the rubber hand, and the critical intervention took place right after the embodiment phase. Participants either kept on moving their hidden real hand and thus the visible rubber hand (active condition), or they stopped moving and kept their hands still (no-movement condition). In a third condition, the rubber hand was struck by a hammer right after the embodiment phase (disruption condition). The rating procedure in the disembodiment phase was the same as in the embodiment phase. The persistence model and the updating model would predict instant disembodiment for the disruption condition because the perceived lack of pain and tactile sensations after seeing the rubber hand being hit by the hammer actively contradicted the rubber hand illusion and updated multisensory bindings. The results confirmed this prediction. For the no-movement condition, the persistence model predicted that embodiment would be maintained because of the absence of sensory signals that might have contradicted the state of embodiment, whereas the updating model predicted continuous, gradual disembodiment. The active condition provided a baseline to evaluate the potential fading of the illusion in the no-movement condition. Indeed, the ratings in the no-movement condition decreased slowly over time. The results therefore favored the updating model, and they are in line with previous studies that demonstrated the importance of multisensory integration for coherent bodily self-perception (e.g., Ehrsson et al., 2004; Gentile et al., 2013; Makin et al., 2008; Newport & Gilpin, 2011).

### The present study

The present study assessed the embodiment and disembodiment of new virtual entities. More specifically, we asked whether embodiment and disembodiment of a 2D virtual hand that could be controlled through movements of a computer mouse or on a touchpad would show temporal dynamics like those demonstrated for a rubber hand (Pfister et al., 2021). Such scenarios appear to be especially relevant for research on disembodiment because the use of tools that mediate our actions in virtual environments has become an essential part of our everyday activities (Haans & IJsselsteijn, 2012; Yee, 2014).

A computer mouse or other device for controlling a cursor on a screen can be considered a special category of tool because they do not directly connect to an object within the physical environment. Instead, they connect physical actions to virtual action effects on the screen (Bassolino et al., 2010; Schettler et al., 2019). One study found that after operating a computer mouse for several minutes, participants extended their peripersonal space to the screen where the virtual interaction, which is controlled by the movements of the mouse, took place (Bassolino et al., 2010). This finding suggests that it is not the computer mouse that is embodied but the cursor, which represents the functional part of the computer mouse within the virtual environment. Interestingly, in a passive condition, it was observed that experienced computer mouse users extended their peripersonal space to the screen after resting their dominant hand on the computer mouse without using it. This indicates that tool-embodiment does not necessarily depend on current sensorimotor experience. Rather, it can occur spontaneously because of extended prior experience with and knowledge about the functional properties of the tool (Schettler et al., 2019).

Thus, several studies have shown that current body representations can be expanded to embody diverse virtual objects ranging from realistic-looking 3D avatars in virtual reality setups to non-corporeal 2D cursers presented on a screen (e.g., Kirsch et al., 2016; Kokkinara & Slater, 2014; Liesner et al., 2020a, b; Maselli & Slater, 2013; Sanchez-Vives et al., 2010; Short & Ward, 2009; Slater et al., 2009). However, it is less clear how these representations evolve after initial embodiment. On the one hand, studies have shown that embodiment of an avatar biases self-perception and behavior even after virtual interaction (Banakou et al., 2013; Yee et al., 2009; Yee & Bailenson, 2007). This suggests that embodiment of virtual entities might sustainably change body representations, and this should be reflected in a relatively slow and prolonged disembodiment. Further support for the assumption of a relatively slow disembodiment of a previously embodied external object comes from findings on the embodiment of non-corporeal objects. Extension of peripersonal space (Farnè & Làdavas, 2000) or a modified body schema (Cardinali et al., 2009; Cardinali et al., 2011) because of active tool use were stable for several minutes after participants stopped using the tool. On the other hand, for subjects who are highly experienced with a tool, the extension of peripersonal space can emerge instantly after grasping the tool even without using it (Bassolino et al., 2010; Serino et al., 2007). This suggests that body representations can change quite dynamically. Thus, if it is assumed that most people have become highly experienced users of a computer mouse, the scenario of a very flexible and fast embodiment and disembodiment in the context of virtual interactions appears plausible as well.

To investigate the temporal dynamics of virtual embodiment and disembodiment, we adapted the paradigm of Pfister et al. (2021) to a virtual setting. Participants controlled a 2D virtual hand by operating a computer mouse or touchpad. To induce embodiment, the virtual hand was repeatedly moved back and forth between two targets (embodiment phase). We then probed for disembodiment in a second phase (disembodiment phase) by comparing two conditions: Participants either continued moving the virtual hand (active condition) or stopped moving and kept their hand still (no-movement condition). Participants rated their sense of embodiment of the virtual hand four times during each phase. Based on previous findings on the disembodiment of a rubber hand (Pfister et al., 2021), we expected ratings to continuously increase during the embodiment phase for both conditions. For the disembodiment phase, we hypothesized that ratings would gradually decrease over time in the no-movement condition but ratings would remain at a constant, high level in the active condition.

## Experiment 1

In Experiment 1, each trial featured a first phase to instill embodiment (embodiment phase) and a second phase to probe for disembodiment (disembodiment phase). During the embodiment phase, participants performed movements with their computer mouse or their touchpad, which were directly transformed into movements of a virtual hand on the participants’ computer screen. During the disembodiment phase, they either continued moving (active condition) or stopped moving (no-movement condition). Embodiment of the virtual hand was measured four times during each phase by asking participants to rate the extent to which the virtual hand felt like a part of their body. We expected embodiment ratings to increase continuously during the embodiment phase for both conditions. For the disembodiment phase, we predicted that embodiment ratings would remain at the level reached during the embodiment phase in the active condition, but we expected embodiment ratings to decrease gradually in the no-movement condition (Abdulkarim et al., 2021; Pfister et al., 2021).

### Method

#### Participants

We collected data from 40 participants and preregistered this sample size (https://www.aspredicted.org/gz8jj.pdf). A power analysis for the contrast probing for disembodiment in the no-movement condition suggested a sample size of 15 participants for a power of 1-β = 0.80 when assuming the effect size of *d*_*z*_ = 0.78, as reported by Pfister et al. (2021). The chosen sample size therefore ensured a high power of 1-β = 0.99 for this effect size while allowing for sufficient power in the face of dropouts and a potentially weaker population effect size. Participants were recruited on the online platform Prolific (https://www.prolific.co/). All experiments reported in this article were conducted according to the ethical regulations of the Ethics Committee of the Institute of Psychology, University of Würzburg.

Following our preregistration, we excluded trials if the embodiment ratings right before the onset of the disembodiment phase were lower than three or if participants moved the virtual hand more than three times during the disembodiment phase of the no-movement condition. We stated in our preregistration that we would also exclude all trials with more than 20 back-and-forth movements between two successive ratings because we supposed that this might be a valid criterion for categorizing movements as too fast. After data collection, however, we found that in most of the trials, participants performed between 20 and 25 back-and-forth movements. Thus, the initial criterion appeared overly conservative, so we relaxed it slightly, considering only trials with more than 25 back-and-forth movements as too fast. We analyzed data only from participants with at least one correct trial per condition. Based on these criteria for trial exclusion, we removed 5 datasets completely as well as 12.9% of the trials (27 in total) for the remaining 35 participants. Of the 27 excluded trials, 21 were due to the embodiment criterion.

One participant did not indicate his age; for the other 34 participants, the mean age was 26.87 years (SD = 6.13; range: 18–46). Of the 31 participants who reported their gender, 20 were male and 11 were female. Of the 32 participants who made statements about their handedness, seven were left-handed, and 25 were right-handed. Five participants made no statement concerning the device they used, 18 participants used a computer mouse, and 12 were equipped with a touchpad. All participants stated that they had fluent English language skills.

#### Apparatus and stimuli

**Fig. 1:**
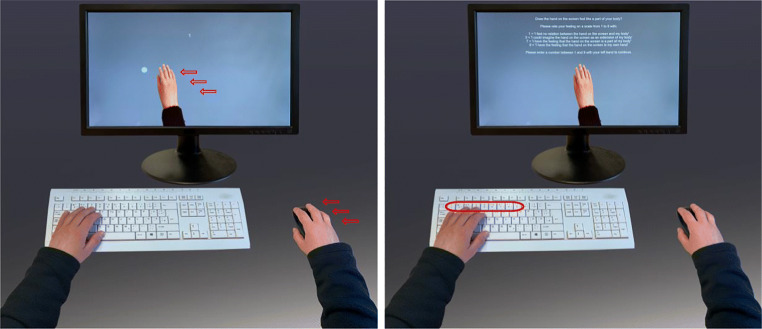
Experimental setup and procedure. *Notes:* Left panel: Participants could control a virtual hand by moving the computer mouse. To instigate embodiment, participants moved the virtual hand toward white target circles that appeared alternatingly on the left and right sides of the display. Reaching the target on one side of the screen made this target disappear and triggered the next target on the opposite side of the screen. Right panel: Participants provided embodiment ratings in regular intervals by using the number keys of the keyboard. Ratings were prompted by a question and verbal anchors for selected rating levels on a scale from 1 to 9 (see text for details). Participants used their right hand to operate the computer mouse and their left hand to enter the numbers for the rating procedure. Because the current study was an online study, the illustrated setup is a prototypical one

Figure 1 illustrates the experimental setup. An image of a right hand was presented on the screen. The overall appearance and orientation of the virtual hand imitated the look of a real hand that controlled a computer mouse or a touchpad. The position was controlled by the x-coordinate of the mouse cursor so that the movements of the computer mouse or touchpad were directly translated onto the virtual hand. Movements along the y-axis did not affect the position of the hand. All stimuli were scaled according to the participant’s display. Two circular targets were presented on the vertical midline with eccentricity of 40% from the midpoint toward either the left or the right edge. The main purpose of the targets was to coordinate the back-and-forth movements of the participants. The virtual hand was aligned to the bottom edge of the monitor, and its size was adjusted to enable exact placement of the virtual index finger on each circular target. Text stimuli during the experiment (e.g., instructions, feedback, or rating questions) were presented in the horizontal center above the virtual hand. The experiment was programmed using PsychoPy (Version v2020.2.8, https://www.github.com/psychopy/psychopy/releases/tag/2020.2.8).

#### Procedure

**Fig. 2:**
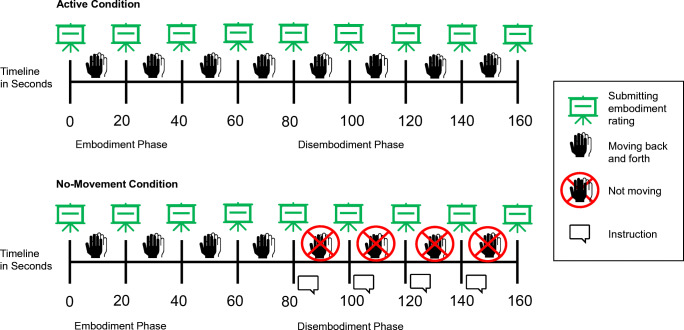
Timeline of events in one trial of either the active or the no-movement condition for Experiment 1. *Notes*: The first embodiment rating was collected before the onset of the trial. Then, the trial started with the embodiment phase. Right after submitting the fifth embodiment rating (after 80 s), the disembodiment phase began. During the disembodiment phase, participants were either supposed to go on moving back-and-forth (active condition) or to stop moving (no-movement condition). Participants rated embodiment of the virtual hand four times in each phase (every 20 s). A more detailed description of the procedure is given in the main text

The exact timeline of events within one trial is shown in Fig. 2 for each condition. In the embodiment phase, participants had to move the virtual hand continuously back and forth from a circular target on one side of the screen to another circular target at the other side of the screen by operating the computer mouse or touchpad with their right hand. Only the currently relevant target area appeared on screen. When the visible target was reached with the virtual index finger, it was immediately replaced by the alternative target. During the whole experiment, if participants moved too fast, they received a warning message (> 18 back-and-forth movements between two successive ratings). The embodiment phase lasted around 80 s, and approximately every 20 s participants rated embodiment of the virtual hand. To prevent interruptions of the back-and-forth movements, the embodiment question was presented upon reaching a target area after at least 20 s had passed since the last rating. The timing was based on previous studies that suggested that embodiment of the virtual hand should emerge within the first minute after the onset of synchronous visuomotor stimulation (Kalckert & Ehrsson, 2017; Pfister et al., 2021).

The critical intervention was implemented in the disembodiment phase. Participants were instructed either to move the virtual hand for another approximately 80 s as in the embodiment phase (active condition) or to stop moving and to keep their hands still for the same amount of time (no-movement condition). In the no-movement condition, no circular targets appeared on the screen. Instead, a brief text was presented above the virtual hand, instructing participants to place the virtual hand in the center of the screen and stop moving. A warning message appeared if the participants did not follow these instructions.

The participants were informed about the experimental procedure in written form in detail before the experiment. The wording of the instruction for the active condition was as follows:

Task 1: If you see a filled circle on the right or left side of the screen, then please move the virtual hand toward this circle until your virtual index finger points directly on the circle. Pointing at a circle will trigger the next target on the opposite side of the screen. The task will consist of several back-and-forth movements from one circular target to the other, and you will be asked a simple question occasionally. Important: In between the questions, you should perform approximately 10–15 back and forth movements. Please move casually from one side to the other as long as you see the circular targets on the screen. Don’t stop moving until you are asked to respond to the question (more on this question on the next screen).

For the no-movement condition, it was:

Task 2: If there are no circular targets on the left or right side of the screen and you see the instruction “Please do not move” instead, then please stay in the screen center and do not move. Important: Please leave your hand on the computer mouse/touchpad and wait until you will be asked the same simple question like in Task 1.

During the disembodiment phase of the no-movement condition, participants received additional brief reminders of the main instructions. For the interval between the last rating of the embodiment phase and the first rating of the disembodiment phase, the wording of this brief reminder was: “Place the hand in the screen center. Then please do not move it.” For the following three inter-rating intervals it was: “Please do not move.” Participants were asked to operate the computer mouse with their right hand.

We asked for embodiment ratings right before the onset of the embodiment phase and approximately every 20 s during the trial – four times in each phase. Thus, we collected nine ratings in each trial. Embodiment ratings were given in response to the question: “Does the hand on the screen feel like a part of your body?” Participants were asked to rate the strength of their feeling on a scale from 1 to 9. Semantic anchors for the rating were provided, as shown in Table 1. Instead of a multi-item questionnaire, which is often used in embodiment studies (e.g., Botvinick & Cohen, 1998), we had a single question to measure embodiment. This allowed us to collect multiple subjective embodiment ratings at different time points without interrupting the ongoing manipulation procedure too much. Participants were instructed to enter the ratings with their left hand and to keep their right hand on the computer mouse or touchpad. Crucially, during the rating procedure, the virtual hand was visible and controllable in the same way as during the tasks between ratings.

**Table 1:** The rating question and semantic anchors used for Experiment 1

Embodiment rating	
Item	Text
Question	Does the hand on the screen feel like a part of your body? Please rate your feeling on a scale from 1 to 9.
Semantic Anchor 1	I feel no relation between the hand on the screen and my body
Semantic Anchor 3	I could imagine the hand on the screen as an extension of my body
Semantic Anchor 7	I have the feeling that the hand on the screen is a part of my body
Semantic Anchor 9	I have the feeling that the hand on the screen is my own hand

A pilot study without specific movement instructions (N = 12) revealed that participants moved excessively fast, with some individuals moving back and forth about 100 times in 20 s. We therefore implemented a movement counter that kept track of the number of back-and-forth movements. Participants were explicitly instructed at the beginning of the experiment to perform 10–15 back-and-forth movements in phases with movement and to move constantly throughout the period between two rating questions. A short break between trials was implemented to disrupt the embodiment of the virtual hand on-screen before the onset of the consecutive trial. During the break, the virtual hand was not visible, and participants were asked to take their hands off the computer mouse or touchpad. After the experiment, the participants were probed about their beliefs regarding the research question in an open-ended debriefing question.

Although the overall procedure was a close replication of previous work on disembodiment (Pfister et al., 2021), there were two notable differences. First, in the present setting, the participant’s real hand was not covered. Second, participants performed hand movements in the horizontal plane and observed the virtual hand moving in the vertical plane. Hence, the orientation of the virtual hand relative to the participants’ body was not fully anatomically plausible. Because these factors have been shown to moderate embodiment of artificial physical or virtual body extensions (e.g., Kalckert & Ehrsson, 2012; Longo et al., 2008; Perez-Marcos et al., 2012; Tieri et al., 2015), we expected to observe lower embodiment ratings than in the previous setup. Therefore, the semantic anchor at rating level three was slightly rephrased from the text used in the earlier study (phrasing in Pfister et al., 2021: “I could imagine that the hand belongs to me”*)* to allow for sufficient variability of responses*.*

All participants were assigned to each condition in a full within-subject design, and they performed three trials of each condition, thus completing six trials in total. The order of conditions was alternated trial by trial, and we randomly assigned whether participants began with the active condition or with the no-movement condition.

### Results

**Fig. 3:**
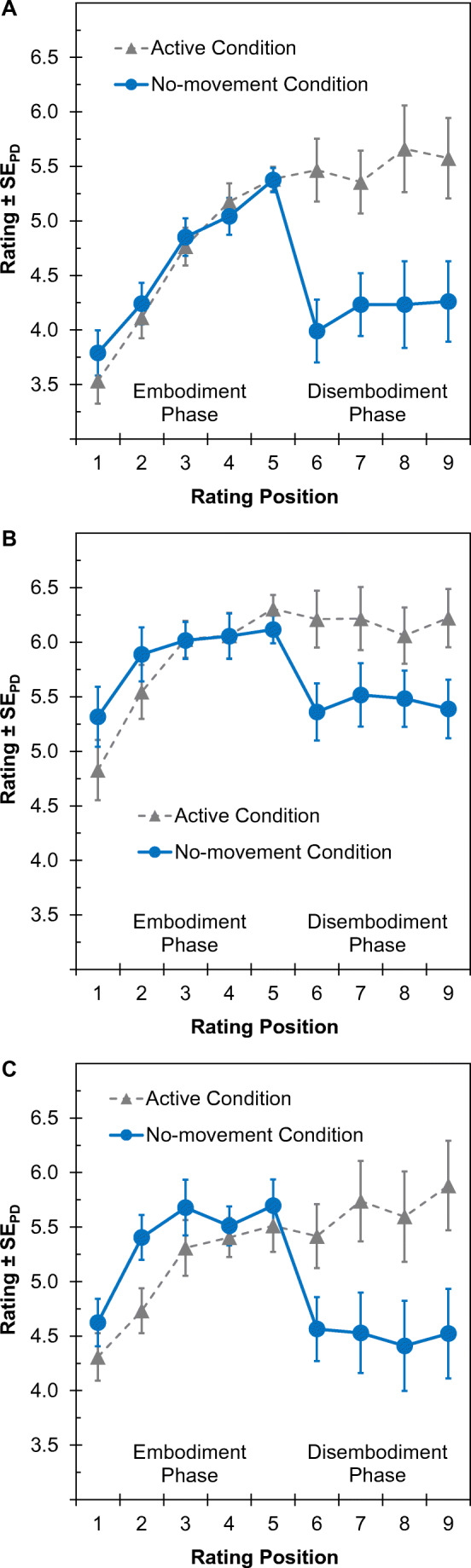
Mean embodiment ratings (scale: 1–9) for the virtual hand during the embodiment phase (rating position: first–fifth) and the disembodiment phase (rating position: sixth–ninth) for Experiment 1 (**a**), Experiment 2 (**b**) and Experiment 3 (**c**). *Notes:* Experimental procedures were identical across both conditions up to the fifth rating. In the active condition (dashed line with triangles), participants continued moving like before, whereas they were instructed to stop moving and keep their hands still in the no-movement condition (solid line with circles). The error bars represent standard errors of paired differences between the two conditions, computed separately for each rating position (SE_PD_; Pfister & Janczyk, 2013)

Figure 3a shows the mean embodiment ratings as a function of rating position (first to ninth) and condition (active vs. no-movement). Raw data and syntax files for recreating the reported analyses and all computer programs and stimulus material for all reported experiments are available online (https://www.osf.io/btcsq/).

#### Analysis plan

The data were analyzed using R, version 4.0.3. All Bayesian analyses were performed using the R package BayesFactor.

For the embodiment phase, we tested the preregistered hypothesis that mean embodiment ratings at the end of the embodiment phase (i.e., for the fifth rating) are higher than mean embodiment ratings at the beginning of the trial (first rating) in a 2 × 2 analysis of variance (ANOVA) with the factors rating position (first vs. fifth rating) and condition (active vs. no-movement). We further probed for equivalence of both conditions by using Bayesian approaches, with BF_01_ > 3.0 as a decision criterion for accepting the null hypothesis of equivalence of both conditions. We had initially preregistered a 2 × 2 Bayesian ANOVA for this purpose. Due to the variability of the corresponding Bayes factor estimates in current implementations of Bayesian ANOVAs (Pfister, 2021), we decided to use Bayesian t-tests to compare embodiment ratings between conditions.

As preregistered, for the disembodiment phase, we expected mean embodiment ratings to evolve differently in the active as compared to the no-movement condition, so we predicted an interaction in a 2 × 2 ANOVA with the factors rating position (fifth vs. ninth rating) and condition (active vs. no-movement). In the next step, we performed a t-test to examine whether mean embodiment ratings would decline in the no-movement condition. A Bayesian t-test was performed to determine whether the mean embodiment ratings remained constant in the active condition. We further planned to assess any gradual decline in the ratings during the disembodiment phase of the no-movement condition by fitting linear versus exponential decay functions.

#### Embodiment phase

A significant main effect of rating position, *F*(1, 34) = 35.07, *p* < .001, η _p_^2^ = .51, indicated that mean ratings increased during the embodiment phase. Neither the main effect of condition, *F*(1, 34) < 1, nor the interaction, *F*(1, 34) = 1.73, *p* = .197, η _p_^2^ = .05, was significant. A Bayesian t-test revealed substantial evidence in favor of the equality of conditions at the end of the embodiment phase, BF_01_ = 5.50. An additional Bayesian t-test was computed to compare the average rating level for the overall embodiment phase between the active and the no-movement conditions. This also supported the null model, suggesting no between-condition differences, BF_01_ = 8.31.

#### Disembodiment phase

The ANOVA revealed a significant main effect of condition, *F*(1, 34) = 12.43, *p* = .001, η _p_^2^ = .27, whereas the main effect of the rating position was not significant, *F*(1, 34) = 3.18, *p* = .084, η _p_^2^ = .09. The predicted interaction was significant, *F*(1, 34) = 10.99, *p* = .002, η _p_^2^ = .24. A follow-up t-test showed that ratings in the no-movement condition declined across the disembodiment phase, *t*(34) = 2.77, *p* = .009, *d*_*z*_ = 0.47. For the active condition, a Bayesian t-test suggested no change of rating over time, BF_01_ = 3.94.

#### Follow-up analyses

Descriptively, the results plotted in Fig. 3a are incompatible with the predicted gradual decline of ratings, as suggested by previous findings on disembodiment of physical entities (Pfister et al., 2021). In contrast, embodiment ratings dropped immediately at the beginning of the disembodiment phase and remained low thereafter. Because of this, we decided to deviate from our analysis plan. Instead, we tested the unpredicted rapid disembodiment in the no-movement condition by contrasting the last rating in the embodiment phase with the first rating of the disembodiment phase (fifth rating vs. sixth rating). The t-test indicated a marked difference for this comparison, *t*(34) = 4.95, *p* < .001, *d*_*z*_ = 0.84. Further, we explored whether the level of the embodiment ratings at the beginning of the disembodiment phase of the no-movement condition was comparable with the level of the embodiment ratings at the end of the trial (sixth rating as compared to the ninth rating). The result favored the null-hypothesis, BF_01_ = 3.61. We further observed an across-participant correlation between the last rating in the embodiment phase (fifth rating) and the difference between this rating and the first rating in the disembodiment phase (fifth rating–sixth rating), *r* = .44, *p* = .008.

### Discussion

The observed increase in embodiment ratings throughout the embodiment phase suggests that participants integrated the virtual hand in their body representation by actively controlling it through movements of a computer mouse or on a touchpad. Surprisingly, however, embodiment ratings dropped considerably already at the beginning of the disembodiment phase of the no-movement condition and did not change thereafter. This suggests that ceasing movement leads to instant disembodiment of the virtual hand.

Prior observations of the disembodiment of a previously embodied physical rubber hand had supported accounts that conceptualize body representation as a set of multisensory bindings (Abdulkarim et al., 2021; Pfister et al., 2021). We took these accounts to expect disembodiment of a 2D virtual hand to unfold steadily over time. Therefore, the instant disembodiment in the no-movement condition is incompatible with our hypothesis. This discrepancy might suggest the involvement of distinct mechanisms in the case of virtual disembodiment.

We discuss this implication in the *General discussion* after addressing methodological limitations that might alternatively explain the current results. In Experiment 2, we ruled out the possibility that the observed rapid disembodiment might reflect anchor effects by removing the critical semantic anchor from the rating scale entirely (e.g., Bishop & Herron, 2015). In Experiment 3, we implemented an attention task during the phases without movement to control the amount of attention participants paid to the virtual environment after ceasing movement. This allowed us to assess whether possible attention shifts from the virtual hand on-screen to the real hand on the computer mouse/touchpad-generated perceptual signals that might have updated the body representation instantly and, hence, led to the rapid disembodiment of the virtual hand on screen (Pfister et al., 2021).

## Experiment 2

The main aim of Experiment 2 was to replicate the unpredicted immediate disembodiment in the no-movement condition and to rule out possible anchor effects. In Experiment 1, the mean embodiment ratings in the disembodiment phase of the no-movement condition dropped instantly to the level of the semantic anchor provided for position 3 of the rating scale (see Table 1). However, rather than indexing the actual disembodiment experience, such a stable response pattern might reflect a tendency to align a response with this semantic anchor. To test this, we removed the anchor in question from the rating scale while retaining all other semantic anchors. If the results of Experiment 1 do indeed reflect subjective embodiment rather than anchoring effects, then the pattern of results should still replicate in this setup.

### Method

#### Participants

On the online platform Prolific, we collected data from 40 participants who had not participated in Experiment 1. Trials were excluded according to the same criteria as in Experiment 1 (for the preregistration of Experiment 2, see: https://www.aspredicted.org/pb595.pdf). This also implies that, as in Experiment 1, we changed the criterion according to which trials were categorized as too fast (more than 25 back-and-forth movements). We analyzed only data from participants with at least one correct trial per condition. Datasets from ten participants had to be excluded, as were 12.8% of the trials for the remaining 30 participants (23 trials in total, 11 of which were due to a lack of embodiment). The mean age of the participants (five female) was 29.3 years (SD = 9.99; range: 18–56). Eight participants were left-handed. Four participants provided no statements about the device they used. Of the remaining 26 participants, 23 used a computer mouse and three a touchpad. All participants stated that they had fluent English language skills.

#### Apparatus, stimuli, and procedure

The apparatus and stimuli were the same as in Experiment 1. The procedure was almost identical, except that we omitted the previously provided anchor for the rating value of three, thus retaining anchors only at positions 1, 7, and 9 of the rating scale.

### Results

Figure 3b shows the mean embodiment ratings plotted for each condition (active vs. no-movement) and rating position (first–ninth). The results of Experiment 2 mirrored the main results of Experiment 1.

#### Embodiment phase

A 2 × 2 ANOVA with the factors rating position (first rating vs. fifth rating) and condition (active vs. no-movement) confirmed the expected main effect of rating position, *F*(1, 29) = 19.25, *p* < .001, η_p_^2^ = .40, but it also revealed an unpredicted interaction, *F*(1, 29) = 5.39, *p* = .027, η_p_^2^ = .16. The main effect of the condition was not significant, *F*(1, 29) < 1. The result of a first Bayesian t-test yielded mixed evidence in favor of the null hypothesis of equality between conditions at the end of the embodiment phase, BF_01_ = 1.92. A second Bayesian t-test indicated that the overall level of embodiment was comparable between conditions across rating positions, BF_01_ = 4.76.

#### Disembodiment phase

Based on the observations of Experiment 1, we preregistered an ANOVA that focused entirely on the disembodiment phase (comparing the sixth and the ninth ratings rather than the fifth and ninth ratings as for Experiment 1). This ANOVA revealed a significant main effect of condition, *F*(1, 29) = 11.75, *p* = .002, η _p_^2^ = .29. Neither the interaction, *F*(1, 29) < 1, nor the main effect of rating position, *F*(1, 29) < 1, was significant. Subsequent Bayesian t-tests provided substantial evidence for the null hypothesis of equality between rating positions in the active condition, BF_01_ = 5.13, and in the no-movement condition, BF_01_ = 5.10.

Relative to the last rating in the embodiment phase, ratings decreased slightly at the first rating position in the disembodiment phase of the active condition, BF_01_ = 1.62. For the no-movement condition, ratings dropped markedly between those two rating positions, *t*(29) = 2.86, *p* = .008, *d*_*z*_ = 0.52.

### Discussion

The results of Experiment 2 replicated the main finding of Experiment 1, despite the changed rating anchors. During the embodiment phase, participants embodied the virtual hand by controlling it through active movements, and they instantly disembodied it after ceasing movement. This finding rules out a possible anchor effect, as the ratings in the disembodiment phase were not close to any remaining anchor.

Close inspection of the chosen experimental design might suggest a second alternative explanation, however. In Experiments 1 and 2, the participants’ real hand that controlled the virtual hand was not covered. After stopping to move the virtual hand, participants might have shifted attention toward their real hand. Attending to the real hand might have contradicted the embodiment of the virtual hand and instantly updated the body representation by rapidly disembodying the virtual hand. Therefore, in Experiment 3, we ensured that participants continuously focused the screen during the disembodiment phase of the no-movement condition with an additional task.

## Experiment 3

To control how much attention participants paid to the virtual hand after they stopped moving it, we implemented an attention task for the disembodiment phase of the no-movement condition. Continuously attending the virtual hand should prevent participants from looking at their real hand and therefore impede potential instant updating of the body representation. If the results of Experiments 1 and 2 were partly due to attention effects, ratings in the disembodiment phase should now decrease gradually. This would be evident in higher ratings at the beginning of the disembodiment phase compared to the last rating. If attention effects did not affect the results of Experiments 1 and 2, then the results of Experiment 3 should replicate the previous findings.

### Method

#### Participants

Using the online platform Prolific again, we collected data from another 40 participants who did not participate in either Experiment 1 or Experiment 2. Besides the criteria used in the preceding experiments for categorizing trials as valid/non-valid, in Experiment 3 trials were excluded if there were two or more mistakes in the attention task (slightly more conservative than preregistered: https://www.aspredicted.org/7862c.pdf). As in the preceding experiments, we categorized trials as too fast if participants made more than 25 back-and-forth movements. For the statistical analyses, we used only data from participants with at least one valid trial per condition. Based on the predefined criteria for trial exclusion we removed 12 datasets entirely. In addition, 17.3% of the trials (29 in total) were excluded for the remaining participants, 23 of which were due to the embodiment criterion. The mean age of the sample was 23.68 years (SD = 5.82, range: 18–47). Out of the 26 participants who reported their gender, 19 were male and seven were female. Twenty-two participants were right-handed, five participants were left-handed, and one participant did not disclose his/her handedness. Four participants participated with a touchpad, 18 with a computer mouse, and six made no statement regarding their technical equipment. All participants stated that they had fluent English language skills.

#### Apparatus, stimuli, and procedure

The apparatus and stimuli were the same as in Experiment 1. Relative to Experiment 1, the only new component of the procedure in Experiment 3 was the attention task in the disembodiment phase of the no-movement condition. In this phase, participants were presented with white circular targets that appeared alternately on the left and right sides of the resting virtual hand. It was randomly selected whether 10, 11, or 12 targets appeared during one inter-rating interval. Participants were instructed to count how many targets appeared after they ceased movement. We tested whether participants engaged in the attention task by asking them after each embodiment rating to state how many targets they had counted during the preceding inter-rating interval.

### Results

Figure 3c displays the results of Experiment 3 as a function of rating position (first–ninth) and condition (active vs. no-movement). The results of Experiment 3 replicated the main findings of the two preceding experiments.

#### Embodiment phase

A 2 × 2 ANOVA with the factors rating position (first rating vs. fifth rating) and condition (active vs. no-movement condition) confirmed the predicted main effect of rating position, *F*(1, 27) = 21.16, *p* < .001, η _p_^2^ = .44. Neither the main effect of condition, *F*(1, 27) = 1.85, *p* = .184, η _p_^2^ = .06, nor the interaction of both factors, *F*(1, 27) < 1, was significant. Although the overall level of embodiment was not equal for both conditions, BF_01_ = 0.06, the mean embodiment ratings right before the onset of the disembodiment phase, at rating position 5, were comparable between conditions, BF_01_ = 3.81.

#### Disembodiment phase

A 2 × 2 ANOVA with the factors rating position (sixth rating vs. ninth rating) and condition (active vs. no-movement condition) yielded a significant main effect of condition, *F*(1, 27) = 11.73, *p* = .002, η _p_^2^ = .30. Neither the main effect of rating position, *F*(1, 27) = 2.15, *p* = .154, η _p_^2^ = .07, nor the interaction between condition and rating position, *F*(1, 27) = 2.81, *p* = .105, η _p_^2^ = .09, was significant. Subsequent Bayesian t-tests comparing the mean embodiment ratings between the sixth and ninth rating positions indicated that the ratings were comparable between the two rating positions in the no-movement condition BF_01_ = 4.89, whereas ratings slightly increased across the active condition, BF_01_ = 0.68.

While embodiment ratings did not change between the fifth and sixth rating positions in the active condition, BF_01_ = 4.25, they decreased markedly in the no-movement condition, *t*(27) = 3.39, *p* = .002, *d*_*z*_ = 0.64. We further computed the percentage of correct responses for the attention task. The result suggested that participants counted the targets quite accurately, as 89.34% of the attention task trials were correct.

### Discussion

The results of Experiment 3 replicated the main findings of Experiments 1 and 2. During the embodiment phase, the participants reported an increasing sense of embodiment of the virtual hand. Embodiment of the virtual hand preserved or even increased further if participants continued moving the virtual hand during the disembodiment phase. However, embodiment ratings immediately dropped if the participants stopped moving the virtual hand. This suggests that the virtual hand was instantly disintegrated from the body representation, although the participants attended the screen continuously. Thus, Experiment 3 showed that attention effects cannot easily account for the observed rapid disembodiment in Experiments 1 and 2.

## General discussion

We conducted three online experiments to investigate how the disembodiment of a previously embodied 2D virtual hand unfolds over time. First, we showed that the virtual hand was embodied after a short period of actively using it. The second and major finding of the current study is that an embodied 2D virtual hand becomes immediately disembodied if participants stop moving it. This unpredicted result suggests that disembodiment in the context of virtual action effects might be especially dynamic and flexible.

### Immediate disembodiment

The immediate disembodiment in the no-movement condition is incompatible with our initial hypothesis, in which we expected that after ceasing movement, disembodiment of the virtual hand would become evident by a gradual decrease of embodiment ratings. This prediction was based on the assumption that the progression of disembodiment of the 2D virtual hand might display a similar temporal dynamic as the disembodiment of a physical rubber hand (Pfister et al., 2021). The results point toward striking differences, however.

According to the terminology proposed by De Vignemont (2011), embodiment of an external object is about processing it in the same way as processing one’s own body. Thus, one might wonder under which circumstances the cognitive system processes an external object as a body part and integrates it in the body representation. The modal view of embodiment attributes changes in bodily self-perception to multisensory integration. In the current study, during the embodiment phase, participants experienced the 2D virtual hand moving synchronously and compatibly with their own hand movements. Thus, based on the integration of correlated visuomotor signals, the cognitive system might have inferred that the virtual hand could be a part of the body (e.g., Kokkinara & Slater, 2014; Sanchez-Vives et al., 2010; Slater et al., 2009). This bottom-up processing of congruent multisensory signals is modulated by top-down knowledge, as is suggested by findings that showed embodiment to vary with the anatomical plausibility of an external object, like humanoid shape (Petkova & Ehrsson, 2008; Tsakiris et al., 2010; Tsakiris & Haggard, 2005) and body continuity (Kalckert & Ehrsson, 2012; Perez-Marcos et al., 2012; Tieri et al., 2015). An external object that looks like a hand and is placed at nearly the same position where one would expect to see his or her own hand would be more likely processed as a part of the body than an external object that does not meet these criteria.

In common physical setups of the moving rubber hand illusion, the rubber hand is placed slightly above the covered real hand, so it looks like it is connected to the rest of the body (Kalckert & Ehrsson, 2014; Pfister et al., 2021). A mechanical connection between the real covered index finger and the corresponding rubber finger allows for an exact spatial and temporal coupling of the real and rubber finger movements. In the current study, participants performed back-and-forth movements with their real hand in the horizontal plane, while the corresponding action effects – i.e., the movement of the 2D virtual hand – were presented in the vertical plane. In addition to this violation of the body continuity constraint, the real hand was not covered. The observation of lower embodiment ratings in the current study (compared to Pfister et al., 2021) might therefore partly reflect different degrees of anatomical plausibility (Maselli & Slater, 2013; Samad et al., 2015).

However, the hypothesis that weak initial embodiment would go along with strong and instant disembodiment is not compatible with the observation of a significant correlation between the last rating in the embodiment phase and the difference between this rating and the first rating in the disembodiment phase of the no-movement condition. This correlation suggests that disembodiment was more rapid for participants who had reported a stronger embodiment experience in the first place. This is the opposite of the assumption that a more rapid disembodiment is associated with a relatively lower level of preceding embodiment. However, the validity of the reported correlation is limited because of the relatively small sample size and the algebraic dependency between the two correlated variables. Thus, the observed correlation should be treated with caution. Future studies are therefore required to address this point directly, for example, by varying the degree of anatomical plausibility between conditions.

Interestingly, research on the embodiment of non-corporeal objects suggests that it is possible to overcome the anatomical plausibility constraint through current or prior experience of actively using the object for an intended interaction (Bassolino et al., 2010; Brugada-Ramentol et al., 2019; Cardinali et al., 2021; Kilteni et al., 2012; Liepelt et al., 2017; Liesner et al., 2021; Ma & Hommel, 2015a, 2015b; Schettler et al., 2019; Serino et al., 2007; Short & Ward, 2009). Thus, based on these findings it could be assumed that not only the embodiment but also the disembodiment of an external object might be due to the amount of agency experience someone has with this object. In this view, the observed rapid disembodiment in the current study possibly reflects that participants are already highly experienced with handling virtual body extensions when interacting with computer technology. In other words, because we spend a great amount of time using different tools for diverse virtual interactions every day, we might have learned to rapidly disembody a virtual body extension as soon as it is no longer needed because task demands have changed. Taken together, the instant disembodiment in the current study possibly suggests that body representations can be flexibly tuned to changing task demands by disembodying parts that are no longer functional for current task performance.

### Implications and future directions

Compared to other studies that induced embodiment of a realistic virtual hand (e.g., Sanchez-Vives et al., 2010), the embodiment of the 2D virtual hand in this study was relatively high. This might further support the assumption that motor control processes play a pivotal role in the formation of body representations. In the current study, the experience of active control or agency might have been critical for the embodiment of the virtual hand because embodiment occurred despite conflicting visuosomatic information of one’s own hand versus the virtual hand, such as displacement and discontinuity. Although several studies have shown that active control over a virtual object promotes embodiment (e.g., Bassolino et al., 2010; Brugada-Ramentol et al., 2019; Liesner et al., 2021; Ma & Hommel, 2015a; Short & Ward, 2009), it is still an unresolved question whether, besides the bottom-up integration of correlated multisensory signals, motor control processes significantly contribute to the embodiment of external objects (e.g., Burin et al., 2015; Burin et al., 2017; Kalckert & Ehrsson, 2012, 2014, 2017; Riemer et al., 2013; Walsh et al., 2011). Regarding the main research question of the current study concerning the disembodiment of external objects, it would be of particular interest to investigate in future studies whether external objects are disembodied differently when preceding embodiment is induced by active movements as compared to passive visuomotor or visuotactile stimulation.

Furthermore, our experimental setup allowed us to assess the time course of embodiment and disembodiment ratings for a remotely controlled 2D virtual hand, highlighting the involvement of actual sensory or sensorimotor input. Here, we add to the relatively sparse literature on the temporal evolution of embodiment sensations (e.g., Ehrsson et al., 2004; Kalckert & Ehrsson, 2017; Pfister et al., 2021). Typically, experiments using bodily illusion report group data only in terms of the strength of the illusion. However, following the time course of these sensations can provide additional and valuable information on the cognitive and neural processes underlying the experience of embodiment of external objects.

The current findings might equally inform applied research, for example, of neuroprosthetic devices (Ehrsson et al., 2008; Velliste et al., 2008) or in digital technologies that aim to manipulate the embodiment experience of the user through basic 2D virtual interactions on-screen or more immersive virtual reality applications (Bohil et al., 2011; Bric et al., 2016; Ortiz-Catalan et al., 2016). First, users may require a certain amount of time until sensations of embodiment develop or fade with the actual sensory information. Second, the experience of active control over a virtual entity that is required for a certain task might promote the embodiment experience and prevent disembodiment of the virtual entity. A better understanding of the processes that enhance or impede embodiment and disembodiment can help to improve the outcomes of therapeutic treatments or training that rely on eliciting embodiment of physical or virtual objects.

### Limitations

Several methodological limitations apply to the present experimental setup. First, it is possible that the chosen time intervals between two ratings (20 s) were not sensitive enough to detect a rapid but gradual decrease in ratings, because such a decay might have occurred within the interval from the no-movement instructions to the first rating of the disembodiment phase. To clarify this point, we plan to test time intervals of different lengths in a future study. This limitation does not undermine our comparison with previous physical setups in which participants reported substantial embodiment through several inter-rating intervals of 30 s each (see also Abdulkarim et al., 2021).

A second limitation stems from the assumption that the disembodiment of a rubber hand and the disembodiment of the 2D virtual hand, which was investigated in the current study, could be directly compared. Our current setup does not allow for any conclusions about whether the 2D virtual hand was experienced as a corporeal or a non-corporeal object. A follow-up experiment comparing the progression of disembodiment between a simple 2D hand and an anatomically more plausible hand could help determine whether the appearance of the virtual object and its location relative to the participant influences the temporal dynamics of disembodiment.

Finally, the validity of the rating scale used in the current study to measure embodiment and disembodiment must be established more thoroughly because it was used in only one other study (Pfister et al., 2021). Assessing how results for this rating scale compare to other questionnaires used in the field is required to increase the comparability to other studies (e.g., Abdulkarim et al., 2021; Dummer et al., 2009; Sanchez-Vives et al., 2010).

### Conclusion

In sum, the current results show that active control of a 2D virtual object that is presented on the computer screen leads to embodiment of the virtual object. The same virtual object becomes instantly disembodied if actively controlling it is no longer required for the current task. The results are consistent with findings showing that the body representation can be extended to integrate tools as a consequence of using them for interacting with other objects in real or virtual environments. The observation that the previously embodied virtual object was disembodied as soon as embodiment was no longer functional for task performance suggests an especially flexible and dynamic adjustment of body representations in the context of virtual interactions.

## Declarations

All reported experiments were conducted according to the ethical guidelines of the German Society of Psychology and the regulations of the local ethics committee of the Department of Psychology at the University of Wuerzburg.

## References

[CR1] Abdulkarim Z, Hayatou Z, Ehrsson HH (2021). Sustained rubber hand illusion after the end of visuotactile stimulation with a similar time course for the reduction of subjective ownership and proprioceptive drift. Experimental Brain Research.

[CR2] Asai T (2016). Agency elicits body-ownership: Proprioceptive drift toward a synchronously acting external proxy. Experimental Brain Research.

[CR3] Banakou D, Groten R, Slater M (2013). Illusory ownership of a virtual child body causes overestimation of object sizes and implicit attitude changes. Proceedings of the National Academy of Sciences.

[CR4] Bassolino M, Serino A, Ubaldi S, Làdavas E (2010). Everyday use of the computer mouse extends peripersonal space representation. Neuropsychologia.

[CR5] Bishop, P. A., & Herron, R. L. (2015). Use and misuse of the Likert item responses and other ordinal measures. *International Journal of Exercise Science, 8*(3), 297–302. https://digitalcommons.wku.edu/ijes/vol8/iss3/10. Accessed 19 Apr 2021 10.70252/LANZ1453 PMC4833473 27182418

[CR6] Blanke O (2012). Multisensory brain mechanisms of bodily self-consciousness. Nature Reviews. Neuroscience.

[CR7] Blanke O, Metzinger T (2009). Full-body illusions and minimal phenomenal selfhood. Trends in Cognitive Sciences.

[CR8] Bohil CJ, Alicea B, Biocca FA (2011). Virtual reality in neuroscience research and therapy. Nature Reviews Neuroscience.

[CR9] Botvinick M, Cohen J (1998). Rubber hands “feel” touch that eyes see. Nature.

[CR10] Bric JD, Lumbard DC, Frelich MJ, Gould JC (2016). Current state of virtual reality simulation in robotic surgery training: a review. Surgical Endoscopy.

[CR11] Brugada-Ramentol V, Clemens I, de Polavieja GG (2019). Active control as evidence in favor of sense of ownership in the moving Virtual Hand Illusion. Consciousness and Cognition.

[CR12] Burin D, Livelli A, Garbarini F, Fossataro C, Folegatti A, Gindri P, Pia L (2015). Are movements necessary for the sense of body ownership? Evidence from the rubber hand illusion in pure hemiplegic patients. PloS ONE.

[CR13] Burin D, Garbarini F, Bruno V, Fossataro C, Destefanis C, Berti A, Pia L (2017). Movements and body ownership: Evidence from the rubber hand illusion after mechanical limb immobilization. Neuropsychologia.

[CR14] Cardinali L, Frassinetti F, Brozzoli C, Urquizar C, Roy AC, Farnè A (2009). Tool-use induces morphological updating of the body schema. Current Biology.

[CR15] Cardinali L, Brozzoli C, Urquizar C, Salemme R, Roy AC, Farnè A (2011). When action is not enough: tool-use reveals tactile-dependent access to body schema. Neuropsychologia.

[CR16] Cardinali L, Zanini A, Yanofsky R, Roy AC, De Vignemont F, Culham JC, Farnè A (2021). The toolish hand illusion: Embodiment of a tool based on similarity with the hand. Scientific Reports.

[CR17] De Vignemont F (2011). Embodiment, ownership and disownership. Consciousness and Cognition.

[CR18] Dummer T, Picot-Annand A, Neal T, Moore C (2009). Movement and the rubber hand illusion. Perception.

[CR19] Ehrsson HH (2009). How many arms make a pair? Perceptual illusion of having an additional limb. Perception.

[CR20] Ehrsson HH, Spence C, Passingham RE (2004). That’s my hand! Activity in premotor cortex reflects feeling of ownership of a limb. Science.

[CR21] Ehrsson HH, Holmes NP, Passingham RE (2005). Touching a rubber hand: Feeling of body ownership is associated with activity in multisensory brain areas. Journal of Neuroscience.

[CR22] Ehrsson HH, Rosén B, Stockselius A, Ragnö C, Köhler P, Lundborg G (2008). Upper limb amputees can be induced to experience a rubber hand as their own. Brain.

[CR23] Farnè, A., & Làdavas, E. (2000). Dynamic size-change of hand peripersonal space following tool use. *NeuroReport, 11*(8), 1645–1649. 10.1097/00001756-200006050-00010 10.1097/00001756-200006050-00010 10852217

[CR24] Folegatti A, de Vignemont F, Pavani F, Rossetti Y, Farnè A (2009). Losing one’s hand: Visual-proprioceptive conflict affects touch perception. PloS ONE.

[CR25] Gallagher S (2000). Philosophical conceptions of the self: Implications for cognitive science. Trends in Cognitive Sciences.

[CR26] Gentile G, Guterstam A, Brozzoli C, Ehrsson HH (2013). Disintegration of multisensory signals from the real hand reduces default limb self-attribution: An fMRI study. Journal of Neuroscience.

[CR27] Haans A, IJsselsteijn WA (2012). Embodiment and telepresence: Toward a comprehensive theoretical framework. Interacting with Computers.

[CR28] Jenkinson PM, Preston C (2015). New reflections on agency and body ownership: The moving rubber hand illusion in the mirror. Consciousness and Cognition.

[CR29] Kalckert A, Ehrsson HH (2012). Moving a rubber hand that feels like your own: A dissociation of ownership and agency. Frontiers in Human Neuroscience.

[CR30] Kalckert A, Ehrsson HH (2014). The moving rubber hand illusion revisited: Comparing movements and visuotactile stimulation to induce illusory ownership. Consciousness and Cognition.

[CR31] Kalckert A, Ehrsson HH (2017). The onset time of the ownership sensation in the moving rubber hand illusion. Frontiers in Psychology.

[CR32] Kilteni K, Normand JM, Sanchez-Vives MV, Slater M (2012). Extending body space in immersive virtual reality: A very long arm illusion. PloS ONE.

[CR33] Kirsch W, Pfister R, Kunde W (2016). Spatial action-effect binding. Attention, Perception, & Psychophysics.

[CR34] Kokkinara E, Slater M (2014). Measuring the effects through time of the influence of visuomotor and visuotactile synchronous stimulation on a virtual body ownership illusion. Perception.

[CR35] Lenggenhager B, Tadi T, Metzinger T, Blanke O (2007). Video ergo sum: Manipulating bodily self-consciousness. Science.

[CR36] Lesur MR, Weijs ML, Simon C, Kannape OA, Lenggenhager B (2020). Psychometrics of disembodiment and its differential modulation by visuomotor and visuotactile mismatches. IScience.

[CR37] Liepelt R, Dolk T, Hommel B (2017). Self-perception beyond the body: The role of past agency. Psychological Research.

[CR38] Liesner M, Kirsch W, Kunde W (2020). The interplay of predictive and postdictive components of experienced selfhood. Consciousness and Cognition.

[CR39] Liesner M, Kirsch W, Pfister R, Kunde W (2020). Spatial action–effect binding depends on type of action–effect transformation. Attention, Perception, & Psychophysics.

[CR40] Liesner M, Hinz NA, Kunde W (2021). How action shapes body ownership momentarily and throughout the lifespan. Frontiers in Human Neuroscience.

[CR41] Longo MR, Schüür F, Kammers MP, Tsakiris M, Haggard P (2008). What is embodiment?. A psychometric approach. Cognition.

[CR42] Ma K, Hommel B (2015). Body-ownership for actively operated non-corporeal objects. Consciousness and Cognition.

[CR43] Ma K, Hommel B (2015). The role of agency for perceived ownership in the virtual hand illusion. Consciousness and Cognition.

[CR44] Makin TR, Holmes NP, Ehrsson HH (2008). On the other hand: Dummy hands and peripersonal space. Behavioural Brain Research.

[CR45] Maravita A, Spence C, Driver J (2003). Multisensory integration and the body schema: Close to hand and within reach. Current Biology.

[CR46] Maselli A, Slater M (2013). The building blocks of the full body ownership illusion. Frontiers in Human Neuroscience.

[CR47] Moseley GL, Olthof N, Venema A, Don S, Wijers M, Gallace A, Spence C (2008). Psychologically induced cooling of a specific body part caused by the illusory ownership of an artificial counterpart. Proceedings of the National Academy of Sciences.

[CR48] Newport R, Gilpin HR (2011). Multisensory disintegration and the disappearing hand trick. Current Biology.

[CR49] Newport R, Pearce R, Preston C (2010). Fake hands in action: Embodiment and control of supernumerary limbs. Experimental Brain Research.

[CR50] Ortiz-Catalan M, Guðmundsdóttir RA, Kristoffersen MB, Zepeda-Echavarria A, Caine-Winterberger K, Kulbacka-Ortiz K, Widehammer C, Eriksson K, Stockselius A, Ragnö C, Pihlar Z, Burger H, Hermansson L (2016). Phantom motor execution facilitated by machine learning and augmented reality as treatment for phantom limb pain: A single group, clinical trial in patients with chronic intractable phantom limb pain. The Lancet.

[CR51] Perez-Marcos D, Slater M, Sanchez-Vives MV (2009). Inducing a virtual hand ownership illusion through a brain–computer interface. NeuroReport.

[CR52] Perez-Marcos D, Sanchez-Vives MV, Slater M (2012). Is my hand connected to my body? The impact of body continuity and arm alignment on the virtual hand illusion. Cognitive Neurodynamics.

[CR53] Petkova VI, Ehrsson HH (2008). If I were you: Perceptual illusion of body swapping. PloS ONE.

[CR54] Pfister R (2021). Variability of Bayes factor estimates in Bayesian analysis of variance. *The Quantitative Methods for*. Psychology.

[CR55] Pfister R, Janczyk M (2013). Confidence intervals for two sample means: Calculation, interpretation, and a few simple rules. Advances in Cognitive Psychology.

[CR56] Pfister, R., Klaffehn, A. L., Kalckert, A., Kunde, W., & Dignath, D. (2021). How to lose a hand: Sensory updating drives disembodiment. *Psychonomic Bulletin & Review*, 1–7. 10.3758/s13423-020-01854-0 10.3758/s13423-020-01854-0 PMC8219564 33300113

[CR57] Ramachandran VS, Rogers-Ramachandran D (1996). Synaesthesia in phantom limbs induced with mirrors. Proceedings of the Royal Society of London. Series B: Biological Sciences.

[CR58] Riemer M, Kleinböhl D, Hölzl R, Trojan J (2013). Action and perception in the rubber hand illusion. Experimental Brain Research.

[CR59] Samad M, Chung AJ, Shams L (2015). Perception of body ownership is driven by Bayesian sensory inference. PloS ONE.

[CR60] Sanchez-Vives MV, Spanlang B, Frisoli A, Bergamasco M, Slater M (2010). Virtual hand illusion induced by visuomotor correlations. PloS ONE.

[CR61] Schettler A, Raja V, Anderson ML (2019). The embodiment of objects: Review, analysis, and future directions. Frontiers in Neuroscience.

[CR62] Serino A, Bassolino M, Farne A, Làdavas E (2007). Extended multisensory space in blind cane users. Psychological Science.

[CR63] Short F, Ward R (2009). Virtual limbs and body space: Critical features for the distinction between body space and near-body space. Journal of Experimental Psychology: Human Perception and Performance.

[CR64] Slater M, Pérez Marcos D, Ehrsson H, Sanchez-Vives MV (2009). Inducing illusory ownership of a virtual body. Frontiers in Neuroscience.

[CR65] Tieri G, Tidoni E, Pavone EF, Aglioti SM (2015). Mere observation of body discontinuity affects perceived ownership and vicarious agency over a virtual hand. Experimental Brain Research.

[CR66] Tsakiris M (2010). My body in the brain: A neurocognitive model of body-ownership. Neuropsychologia.

[CR67] Tsakiris M, Haggard P (2005). The Rubber Hand Illusion revisited: Visuotactile integration and self-attribution. Journal of Experimental Psychology: Human Perception and Performance.

[CR68] Tsakiris M, Prabhu G, Haggard P (2006). Having a body versus moving your body: How agency structures body-ownership. Consciousness and Cognition.

[CR69] Tsakiris M, Carpenter L, James D, Fotopoulou A (2010). Hands only illusion: Multisensory integration elicits sense of ownership for body parts but not for non-corporeal objects. Experimental Brain Research.

[CR70] Vallar G, Ronchi R (2009). Somatoparaphrenia: A body delusion. A review of the neuropsychological literature. Experimental Brain Research.

[CR71] Velliste M, Perel S, Spalding MC, Whitford AS, Schwartz AB (2008). Cortical control of a prosthetic arm for self-feeding. Nature.

[CR72] Walsh LD, Moseley GL, Taylor JL, Gandevia SC (2011). Proprioceptive signals contribute to the sense of body ownership. The Journal of Physiology.

[CR73] Yee, N. (2014). *The Proteus paradox: How online games and virtual worlds change us–and how they don’t*. ProQuest Ebook Central. https://www.ebookcentral.proquest.com/lib/ub-wuerzburg/detail.action?docID=3421350. Accessed 14 June 2021

[CR74] Yee N, Bailenson J (2007). The Proteus effect: The effect of transformed self-representation on behavior. Human Communication Research.

[CR75] Yee N, Bailenson JN, Ducheneaut N (2009). The Proteus effect: Implications of transformed digital self-representation on online and offline behavior. Communication Research.

